# RETRACTION: Female Lizards (*Eremias Argus*) Reverse Bergmann’s Rule Across Altitude

**DOI:** 10.1002/ece3.11683

**Published:** 2024-07-12

## Abstract

**RETRACTION**: G. G. Deme, X. Liang, J. O. Okoro, P. Bhattarai, B. Sun, Y. D. Malann, and R. A. Martin, “Female Lizards (*Eremias Argus*) Reverse Bergmann’s Rule Across Altitude,” *Ecology and Evolution* 13, no. 8 (2023): e10393, https://doi.org/10.1002/ece3.10393.

The above article, published online on 07 August 2023 in Wiley Online Library (wileyonlinelibrary.com), has been retracted by agreement between the authors; the journal Editors‐in‐Chief, Allen Moore, Andrew Beckerman, Gareth Jenkins and Marcus Lashley; and John Wiley & Sons Ltd. The authors asked to retract the article as they found significant errors in the dataset used for the analyses presented in the article and publicly archived on Dryad (https://doi.org/10.5061/dryad.41ns1rnkm). The dataset contains the same value of female snout‐vent length (53.21mm) repeated across 138 rows for two populations (Gonghe and Erdos). Following the authors’ request to retract, further concerns were raised regarding additional anomalies in the dataset and inconsistencies in the used models and the presented data analysis, therefore the results and the conclusions of the paper are not supported.

**RETRACTION**: G. G. Deme, X. Liang, J. O. Okoro, P. Bhattarai, B. Sun, Y. D. Malann, and R. A. Martin, “Female Lizards (*Eremias Argus*) Reverse Bergmann’s Rule Across Altitude,” *Ecology and Evolution* 13, no. 8 (2023): e10393, https://doi.org/10.1002/ece3.10393.

The above article, published online on 07 August 2023 in Wiley Online Library (wileyonlinelibrary.com), has been retracted by agreement between the authors; the journal Editors‐in‐Chief, Allen Moore, Andrew Beckerman, Gareth Jenkins and Marcus Lashley; and John Wiley & Sons Ltd. The authors asked to retract the article as they found significant errors in the dataset used for the analyses presented in the article and publicly archived on Dryad (https://doi.org/10.5061/dryad.41ns1rnkm). The dataset contains the same value of female snout‐vent length (53.21mm) repeated across 138 rows for two populations (Gonghe and Erdos). Following the authors’ request to retract, further concerns were raised regarding additional anomalies in the dataset and inconsistencies in the used models and the presented data analysis, therefore the results and the conclusions of the paper are not supported.

